# The Thai Health Promotion Foundation: Two Decades of Joint Contributions to Addressing Noncommunicable Diseases and Creating Healthy Populations

**DOI:** 10.9745/GHSP-D-23-00311

**Published:** 2024-04-29

**Authors:** Viroj Tangcharoensathien, Supreda Adulyanon, Nuttapun Supaka, Rungsun Munkong, Shaheda Viriyathorn, Siriya Sirithienthong, Siriyaporn Kanhachon, Robert Marten

**Affiliations:** a International Health Policy Program, Ministry of Public Health, Nonthaburi, Thailand.; b Thai Health Promotion Foundation, Bangkok, Thailand.; c Alliance for Health Policy and Systems Research, World Health Organization, Geneva, Switzerland.

## Abstract

Globally, the current investment in preventive care is inadequate and ineffective for addressing noncommunicable diseases and their causes. The Thai Health Promotion Foundation, with its sustainable funding from 2% levies on tobacco and alcohol, together with partners, has been used to address noncommunicable diseases effectively.

## Key Messages

- Despite the global burden of noncommunicable diseases (NCDs) and evidence that investment in preventive care improves health and is cost effective, per capita spending on preventive care is very low compared to curative care.
- Prioritizing preventive care strategies to address NCD risk factors requires strong government leadership, supportive legislation and health policies, and multisector interventions.
- Health promotion foundations that have secure and sustainable funding can be used strategically to address NCD risk factors, complement government spending on preventive care, and contribute to promoting healthy populations.
- Together with partners, the Thai Health Promotion Foundation, established in 2001 and funded by a 2% levy on tobacco and alcohol, contributed significantly to addressing NCD risk factors. It can serve as a model for other countries.

## INTRODUCTION

In light of the ongoing noncommunicable disease (NCD) epidemic and slow progress in addressing NCDs—despite several United Nations General Assembly High-Level Political Declarations since 2011^1^^,^^2^—we argue that existing investments in health promotion are inadequate and not effective to address NCDs and create healthy populations. We showcase the Thai Health Promotion Foundation (ThaiHealth) as an example of how an institution can serve as a model for other countries considering how to address NCDs and create healthy populations.^3^

In this article, we refer to the top 10 causes of death in Thailand, which cover 8 NCD conditions^4^: ischemic heart disease, stroke, chronic kidney disease, liver cancers and cirrhosis, lung cancer, Alzheimer’s disease, and diabetes. Note that mental health is not among the top 10 causes of mortality. The top 5 risk factors that contributed to disability-adjusted life year loss in 2019 were tobacco use, high fasting plasma glucose, high body mass index, high blood pressure, and alcohol use. All of these are in the ThaiHealth priority program of work.

Despite the evidence that investing in prevention improves health, is cost effective, and promotes and stimulates economic growth,^5^ the majority of health spending is for treating diseases rather than investing in preventing health risks and creating healthy populations. A study of national health accounts from 195 countries showed that, of the total estimated US$1019 per capita current health expenditures in 2017, 51.3% and 17.5% were spent on curative care and medical goods, while only 4.6% was for prevention.^6^ More broadly, strategies to address NCDs should go beyond a narrow “medicalized framing” of the NCD problem and its solutions.^7^

We analyzed World Health Organization (WHO) global health expenditures in 134 countries where spending on preventive care was available (Table 1). Despite great variation across country income status, the global average preventive care expenditure was US$61.70 per capita or 8.7% of current health expenditure in recent years. Donor resources play a significant role in financing prevention in low- and lower-middle-income countries.

**TABLE 1.** Expenditure on Health Prevention by Country Income Group, Global Analysis, Latest Available Year, 2021

	**Average Preventive Care**		**Source of Preventive Care Expenditure, %**		
	**US$ per Capita**	**% of Current Health Expenditure**	**Government**	**Donor**	**Nongovernment**
High	154.4	4.3	91.7	0.3	8.0
Upper-middle	25.0	6.1	79.7	8.7	11.6
Lower-middle	10.9	11.3	44.6	43.6	11.8
Low	6.6	17.9	15.3	77.6	7.1
All income groups	61.7	8.7	58.7	31.3	10.0

Source: Authors’ analysis from WHO Global Health Expenditure database: https://apps.who.int/nha/database/ViewData/Indicators/en.

Analysis of Thailand’s budget allocation for prevention and health promotion from the National Health Security Office shows that almost all the investments are for clinical preventive services. This includes maternal and child health services (e.g., antenatal and postnatal care); family planning and vaccination services; secondary prevention (e.g., screening for Down’s Syndrome, congenital hypothyroidism, and cervical cancers); and priority health problems in local communities, provinces, and regions. Though some of the National Health Security Office’s supported “community health fund” with equal matching budget from the local government are used for health promotion campaigns to address local NCD challenges, the per capita budget for prevention and health promotion is less than one-third of outpatient care. On average, the budget approval for prevention and health promotion between 2010 and 2021 was US$9.30 per capita population (Table 2).

**TABLE 2.** Thailand’s Universal Coverage Scheme Budget Allocation, 2010–2021^a^

**Fiscal Year**	**Universal Coverage SchemeBudget Approved**	**OutpatientCare**	**InpatientCare**	**Prevention and HealthPromotion**
2010	80.0	25.2	29.8	6.6
2011	84.9	26.5	31.8	7.2
2012	91.9	32.9	32.4	7.8
2013	91.9	32.8	32.5	7.7
2014	96.5	35.2	34.3	9.6
2015	96.5	35.2	33.3	9.5
2016	101.0	36.8	35.3	9.9
2017	103.7	37.9	36.4	10.1
2018	109.4	38.9	40.0	10.3
2019	114.2	39.7	43.2	10.6
2020	120.0	41.7	45.7	11.0
2021	124.0	42.7	48.0	11.0
Average 12 years	101.2	35.5	36.9	9.3

^a^ Per capita budget in US$ was calculated using 30 Baht exchange rate for all years.

Source: National Health Security Office budget approval database.

In Thailand, the per capita budget for prevention and health promotion is less than one-third of outpatient care.

## EFFECTIVENESS OF HEALTH PREVENTION PROGRAMS

The U.S. National Commission on Prevention Priorities recommended 3 highest-ranked, cost-effective clinical preventive services, including counseling aspirin use with high-risk adults, immunizing children, and tobacco-use screening and brief intervention. However, implementing these recommended services needs to address its low coverage (less than 50%).^8^ In implementing the Affordable Care Act in the United States, efforts are underway to increase awareness of providers, insurance plans, and patients to increase utilization and avoid unnecessary bills to patients for these clinical preventive services.^9^ The greatest benefit could be achieved by increasing the coverage of clinical preventive services that address tobacco use, obesity-related behaviors, alcohol misuse, colorectal cancer, and influenza.^10^

To achieve health gain beyond individuals, a proposed framework for prioritizing clinical preventive services can be applied to community-based interventions and other health care service settings.^11^ These cost-effective clinical preventive services also should be extended to community-based interventions^12^ by addressing the determinants of health, notably, the physical and social environments, such as obesogenic^13^ and alcogenic environments.^14^ Alcogenic environments include settings with unregulated advertising and marketing of alcoholic refreshments, higher liquor outlet density, products planned to facilitate affordability, and low prices of alcoholic refreshments.^15^ Effective obesity prevention programs should combine person-based determinants and address obesogenic environments.^16^ Interventions targeting built and social environments promote physical activity effectively.^17^^,^^18^ Evidence from a Cochrane systematic review suggests that physical activity reduces mortality rates and improves quality of life with minimal or no safety concerns.^19^

Further, clinical preventive services and community-based interventions need to be fostered by macro-level policy, legislation, and regulation that address key health determinants, such as tobacco, alcohol, and unhealthy diet. Evidence shows that implementing the WHO “Best Buy” interventions on NCDs not only saves millions of lives but also results in a return on investment of US$7 for every US$1 invested.^20^ Effective implementation of these interventions needs to address key barriers, such as policy incoherence across government sectors,^21^ policy interference by industries,^22^ cross-border marketing,^23^ conflicts of interest,^24^ and implementation capacity.^25^

The most effective preventative strategies are changes in diet, increases in physical activities, and the cessation and reduction of tobacco and alcohol use.^26^ Health risks include both proximal and distal causes. Distal socioeconomic factors include income, education, and occupation, all of which determine physical activity, diet, tobacco, and alcohol use. These interact with physiological and pathophysiological causes, such as blood pressure, cholesterol levels, and glucose metabolism, resulting in cardiovascular disease and stroke.^27^

Population-wide prevention interventions result in greater health outcomes than screening and treatment of individuals at risk. Shifting the mean systolic blood pressure in the whole population is more cost effective in preventing heart attacks and strokes.^28^ For example, in Finland, a 10% reduction in cholesterol levels across the population would reduce coronary heart disease mortality by 20%.^29^ Dietary changes, reduced smoking in males, and better blood pressure control contributed to Finland’s dramatic decline in coronary heart disease mortality.^30^

Population-wide prevention interventions result in greater health outcomes than screening and treatment of individuals at risk.

## BEST BUY INTERVENTIONS: STRONG LEADERSHIP IS REQUIRED

Most WHO Best Buy interventions (measured by a cost-effectiveness threshold of less than 100 international dollars per disability-adjusted life year averted in low- and lower-middle-income countries) lie outside the health sector, requiring leadership and multisectoral actions for health. These interventions include, among others, increasing taxes and prices on tobacco and alcohol, implementing standardized tobacco packaging and large graphic health warnings, banning tobacco advertising, restricting the availability of alcohol, and eliminating exposure to secondhand tobacco smoke.^31^ Increasing taxes on unhealthy products not only reduces their consumption but also generates revenues that can be invested in creating healthy populations.

Legislating and enforcing these interventions requires strong government leadership, particularly against political interference by tobacco, alcohol, and soft drink industries,^32^ as well as sustainable financing to advocate for and create public awareness.^33^ Moreover, implementing these interventions requires countries to have a strong capacity to identify and adapt interventions to their local context.^34^

## HEALTH PROMOTION FOUNDATIONS: A POTENTIAL SOLUTION

The Health Promotion Foundation Act in 2001 established ThaiHealth and provided legal mandates to promote health aligned with national health policy, develop health promotion capacity in communities, support research and generate evidence to support health promotion, and advocate for public awareness on NCD risk factors, such as alcohol and tobacco use. To fulfill these legal responsibilities, the Act empowers ThaiHealth to collect a 2% levy on tobacco and alcohol excise tax.

ThaiHealth is chaired by the Prime Minister, with the Public Health Minister as the first vice chair and an expert as the second vice chair. The Cabinet appoints 9 ex officio members from relevant ministries and 8 experts from the fields of health promotion, community development, public communication, education, sports, culture, laws, and administration.

To ensure accountability, Article 36 of the Act mandates that ThaiHealth submits an annual report that includes performance and audited financial statements to the Cabinet, House of Representatives, and the Senate for consideration. The annual report is made publicly available on its website to ensure transparency. To provide oversight on ThaiHealth’s performance, Article 37 of the Act establishes an Evaluation Committee with a chair and 6 experts in the fields of finance and health promotion with at least 2 evaluation experts. Experts are appointed by the Cabinet as proposed by the Minister of Finance. Finally, Article 40 of the Act enforces the payment of the levy. Any tobacco and alcohol importers and manufacturers that fail to pay are subject to no more than 1 year of imprisonment and/or a financial penalty of five- to twentyfold the amount liable to ThaiHealth.

### Revenue and Expenditure

We showcase and analyze revenue and expenditure profiles of ThaiHealth funds from 12 years. Between 2010 and 2021, the average annual revenue from the 2% tobacco and alcohol surcharge was US$131.0 million, of which US$88.8 million (67.8%) was from alcohol and US$42.2 million (32.2%) from tobacco. The per capita Thai population contribution to ThaiHealth revenue was small (US$2.00.) Revenue peaked at US$146.7 million in 2017 and reduced slightly during the COVID-19 pandemic in 2020–2021 to US$136.5 million in 2021 (Table 3).

**TABLE 3.** ThaiHealth Annual Revenue, 2010–2021^a^

**Fiscal Year**	**Revenue, US$ Million**	**From Tobacco, US$ Million**	**From Alcohol, US$ Million**	**Revenue, US$ per Capita Population**
2010	103.7	35.8	67.9	1.6
2011	113.1	38.6	74.4	1.8
2012	118.7	39.9	78.8	1.8
2013	127.1	42.9	84.2	2.0
2014	135.5	43.8	91.7	2.1
2015	137.5	40.1	97.4	2.1
2016	142.9	44.9	98.0	2.2
2017	146.7	47.5	99.2	2.2
2018	134.7	46.2	88.5	2.0
2019	139.0	41.7	97.3	2.1
2020	136.5	43.3	93.2	2.1
2021	136.5	42.0	94.5	2.1
Average 12 years	131.0	42.2	88.8	2.0

^a^ Revenue in US$ was calculated by 30 Baht to US$ exchange rate throughout 12 years.

Source: Total ThaiHealth revenue was analyzed from ThaiHealth annual reports 2010–2021: https://bit.ly/3KGpAs2; proportion of revenue from tobacco, alcohol (liquor + beer) from Ministry of Finance: https://bit.ly/3Y7zKF3.

Table 4 shows how this revenue was used and invested to support both government and nongovernmental organizations between 2010 and 2021. The large share was strategic funding support to address NCD risks—including tobacco, alcohol, traffic injuries, diet, and physical activities—through direct programmatic campaigns and strategic movement to support legislation and regulation, generate evidence, and inform policies. Between 2010 and 2021, ThaiHealth invested 32.1% of the average annual total of US$121.4 million to address NCD risk factors. Note that unspent funding, compared with total annual revenue, was carried over to the next fiscal year and remained in the ThaiHealth Fund.

**TABLE 4.** ThaiHealth Total Funding Support by Funding Categories, 2010–2021

**Fiscal Year**	**Total Funding Support, US$ Million**	**Distribution by Funding Categories, %**			
		**Noncommunicable Disease Risk Factors^a^**	**Healthy Community Strengthening**	**Health Literacy, Healthy Media System**	**Others**
2010	90.1	30.0	10.4	0.0	59.6
2011	92.0	27.2	13.6	0.6	58.6
2012	106.6	28.6	14.1	15.2	42.1
2013	104.5	29.8	14.5	3.8	51.9
2014	153.3	31.7	11.0	15.1	42.3
2015	159.8	29.1	16.1	16.0	38.8
2016	130.4	34.0	14.1	12.3	39.6
2017	147.2	30.7	11.8	14.9	42.6
2018	131.0	36.7	11.4	12.9	38.9
2019	129.2	32.6	11.3	19.0	37.2
2020	107.5	35.2	10.0	14.4	40.4
2021	105.8	39.8	9.0	12.0	39.2
Average 12 years	121.4	32.1	12.3	11.3	44.3

^a^ Program for noncommunicable disease risk factors includes tobacco, alcohol, traffic injury prevention, promotion of physical activity, and healthy diet.

Source: Thai Health Promotion Foundation. Annual Report Thai Health Promotion Foundation: https://bit.ly/3KGpAs2.

### Examples of Major Contributions to Creating Healthy Populations

ThaiHealth has supported the creation of evidence and the advancement of broader policies to control NCD risk factors and create healthy populations.

ThaiHealth has supported the creation of evidence and the advancement of broader policies to control NCD risk factors and create healthy populations.

In 2008, ThaiHealth and partners provided evidence for legislation of the Alcoholic Beverage Control Act, the first comprehensive law that banned advertising and promotions, regulated the minimum legal drinking age of 20 years, created new warning labels, and restricted hours of alcohol sale.

In 2017, ThaiHealth and partners supported evidence for legislating the Tobacco Product Control Act, which applied the WHO Best Buy interventions on reducing tobacco use by placing a large pictorial warning on the front of cigarette packs, extending smoke-free areas to all public places, implementing a total ban of tobacco advertisements, and banning the import and sale of all types of electronic nicotine delivery systems despite aggressive industry interference.^35^ At the same time, the Ministry of Finance also increased the tobacco excise tax from 75% in 2001 to 87% in 2012, in line with the WHO recommendation. The Thailand government’s efforts to raise tobacco tax and ban electronic nicotine delivery systems were interfered with by tobacco industries using various tactics such as creating front groups, lobbying decision-makers, running public relations campaigns, seeking to discredit tobacco control advocates, and funding pro-tobacco harm reduction research.^35^

ThaiHealth and partners provided evidence for legislating the Control of Marketing of Infant and Young Child Food Act in 2017, which bans the advertising of infant formula milk.^36^ In 2018, ThaiHealth supported evidence for introducing taxes on sugar-sweetened beverages and banning trans fat in domestic and imported food products.^37^ Alongside this, ThaiHealth and nongovernmental organizations advocated and supported the implementation of a zero-sugar drink policy in thousands of Thai schools.

The Ministry of Public Health endorsed the first National Physical Activity Plan (2018–2030)^38^ with active support from ThaiHealth and key domestic and international partners. ThaiHealth and partners proposed a 2016 South East Asia Regional Committee resolution, “Revitalizing Physical Activity,” which was adopted by the Regional Committee. Related to this, ThaiHealth seconded a scientist to work with WHO in the development of the Global Action Plan on Physical Activity (2018–2030), which was adopted by the 71st World Health Assembly in 2018.

### Contributions to Improved Noncommunicable Disease Outcomes

The establishment of ThaiHealth coincided with improvements in NCD risk factors. The prevalence of adult smoking decreased from 25.5% in 2001 to 17.4% in 2021, and the prevalence of alcohol drinking in adults decreased from 32.7% in 2004 to 28.0% in 2021. Sufficient physical activity increased from 66.3% in 2012 to 74.6% in 2019 but dropped to 57.5% in 2021 due to the impacts of public health and social measures during the COVID-19 pandemic.^39^ Further, the number of road traffic fatalities declined from 21,996 in 2011 to 16,957 in 2021.^40^ In fact, Thailand ranks first in the South East Asia Region on the effective implementation of WHO Best Buy interventions by fully achieving 13 and partially achieving 6 indicators.^41^

NCDs continued to be the leading cause of death in the Thai population in 2009 and 2019. Eight of the top 10 leading causes in both years were NCDs, with the death rate per 100,000 increasing between 2009 and 2019 (Table 5).^4^

At best, ThaiHealth and its partners contributed to the reduction of risk exposure; the mortality cannot be reversed in the short run. NCD conditions are the outcomes of long-term exposure to risk factors, especially smoking, hypertension, and overweight, as reported by the Rotterdam cohort study.^42^

In this analysis, we cannot establish ThaiHealth’s attribution to the reduction of NCD risk factors (e.g., tobacco and alcohol use). We also refrain from making such an attribution because other partners, particularly the Ministry of Public Health and the Department of Excise that were responsible for increasing the tobacco and alcohol tax as well as civil society organizations, have played roles in the reduction. ThaiHealth and other partners successfully developed multisectoral collaboration based on mutual recognition and respect of joint contributions with a common shared goal. Other factors have hampered the progress in other areas, such as reducing road traffic mortality, including low awareness of the mandatory helmet law^43^ and poor enforcement of drunk driving. Thailand’s self-assessment scored 6 of 10 on drunk driving enforcement.^44^

Although these institutions are not a “magic bullet” and do not singlehandedly lead to improved outcomes, they can be an important part of a broad public health strategy. Of course, investments from health promotion foundations need to be aligned across the government with strong community engagement. For example, a study in 5 high-income countries suggested funding from health promotion foundations has the most impact when aligned with other governance mechanisms as part of a broader investment to create healthy populations.^45^

## DISCUSSION

Various publications have provided insights into different dimensions of ThaiHealth’s efforts and a review of its first decade of work.^46^^–^^48^ This article offers several new contributions by analyzing average global per capita spending on preventive care and sources of finance, the National Health Security Office per capita spending on health promotion and prevention, and providing an update on ThaiHealth’s revenue generated from tobacco and alcohol and on NCD outcomes in Thailand. Further, we provide additional insights into the unique attributes of ThaiHealth that could provide useful lessons for other countries.

First, ThaiHealth fosters social transformation conducive to health through the creation of a culture where healthy lifestyles are seamlessly integrated into the daily lives of people from all levels of society. The different sugar-sweetened beverage tax rates based on sugar content have reoriented the beverage industry to produce less sugar content, which benefits population health, makes healthier choices more accessible, affordable, and socially desirable, and empowers citizens through community radio networks.

Second, despite its small annual expenditure of US$121.4 million (Table 4), ThaiHealth’s strategic focus on addressing the priority social and commercial determinants of health has made a significant difference in improving NCD outcomes.

**TABLE 5.** Top 10 Causes of Deaths per 100,000 Population in 2019 and Mortality Rate Change Between 2009 and 2019, All Ages Combined, Thailand

**Cause** ^a^	**2009 Rank**	**2019 Rank**	**Change in Deaths per 100,000,2009 to 2019, %** ^b^
1. Ischemic heart disease	2	1	17.4
2. Stroke	1	2	14.0
3. Lower respiratory infection	5	3	18.5
4. Chronic kidney disease	4	4	12.3
5. Liver cancer	6	5	9.3
6. Lung cancer	8	6	7.4
7. Alzheimer’s disease	11	7	12.4
8. Cirrhosis of liver	10	8	5.5
9. Diabetes	12	9	9.7
10. Road injuries	3	10	−1.7

^a^ All are noncommunicable diseases except lower respiratory disease and road injuries.

^b^ Change in deaths per 100,000 between 2009 and 2019 was adjusted by using global standardized age and sex. It considers population growth and aging.

Source: The Institute for Health Metrics and Evaluation: https://www.healthdata.org/research-analysis/health-by-location/profiles/thailand.

ThaiHealth’s strategic focus on addressing the priority social and commercial determinants of health has made a significant difference in improving NCD outcomes.

Third, health promotion spending by universal health coverage systems and the Ministry of Public Health focuses on clinical preventive services, but ThaiHealth has effectively filled the gaps through broad-based citizen engagement.

Finally, over the past 2 decades, ThaiHealth’s social capital has been strengthened by implementing broad-based citizen engagement that focused on vulnerable populations affected most by commercial marketing and working for vulnerable populations through civil society organizations, such as community-based HIV programs, multisectoral governance, and transparent budget allocation. Despite bureaucratic politics and the introduction of a maximum of 4 billion Thai Baht annual revenue from tobacco and alcohol surcharge (which failed to amend the ThaiHealth Act in 2017),^49^ ThaiHealth has managed to survive because civil society organizations mobilized active citizens to protect it from political interference by these bureaucrats and interference by tobacco and alcohol industries.

## CONCLUSION

ThaiHealth strategic financing, though comparatively small, contributes to leveraging various legislative and regulatory measures that apply WHO Best Buy interventions against NCDs and contribute to creating healthy societies. While preventive medical services financed by a universal health coverage system are essential for different populations, secure and sustainable financing for broader public health and population-based approaches to address NCD risk factors, strengthen communities, build health literacy, and promote health are equally important. As demonstrated by ThaiHealth, health promotion foundations, funded by health taxes on products like tobacco and alcohol, can significantly accelerate efforts to create healthy populations.

## Notes

**Peer Reviewed**

**First published online:** March 6, 2024.
